# Pathways to practice: Structural determinants of internationally educated nurses’ transition into the U.S. healthcare workforce

**DOI:** 10.1016/j.jnr.2026.07.001

**Published:** 2026-08-06

**Authors:** Maria Luisa B. Ramira, Mary Joy Garcia-Dia, Reynaldo R. Rivera, Carmina T.V. Bautista, Jacqueline Juele-Schuster, Lorraine S. Evangelista, Marife Sevilla

**Affiliations:** a Nursing Faculty, Southwestern College Higher Education, San Diego, California, USA; b Associate Adjunct Faculty, Case Western Reserve University, Frances Payne Bolton School of Nursing, Cleveland, Ohio, USA; c Director of Nursing Research and Innovation, New York-Presbyterian Hospital and Assistant Clinical Professor of Nursing, Columbia University School of Nursing, New York, New York, USA; d Chair PNAA Business Development, North Brunswick, New Jersey, USA; e New York City Hospitals, Certified Wound Specialist, New York, New York, USA; f Professor Emerita, Sue & Bill Gross School of Nursing, University of California Irvine, Irvine, California, USA; g Chair PNAA Human Rights Committee, North Brunswick, New Jersey, USA

**Keywords:** Internationally educated nurses, Global nursing workforce, Ethical recruitment, Predeparture preparation, Health workforce policy, Workforce transition

## Abstract

**Background:**

Nurse migration can create opportunities for career growth and financial stability; however, these nurses may face challenges related to cultural adjustment, workplace integration, and recruitment and employment practices.

**Purpose:**

To describe the career pathways of internationally educated nurses (IENs) in the United States with a focus on the recruitment process and transition of newly hired nurses into the healthcare system.

**Methods:**

A cross-sectional survey design was employed to understand the demographic and professional characteristics of IENs and to assess structural factors influencing the transition to practice, including recruitment, predeparture preparations, and support received during the transition.

**Results:**

Data showed that IENs experienced varying levels of support across recruitment, pre-arrival preparation, arrival, and onboarding. Travel and relocation assistance were commonly reported, whereas structured pre-arrival preparation, formal IEN orientation, cultural orientation, and ongoing follow-up were less consistent. These differences highlight opportunities to strengthen transition support across the pathway to practice.

**Conclusion:**

Interrelated structural processes, rather than individual adaptation alone, shape the integration of IENs into U.S. nursing practice. These findings highlight the important role of nursing regulation in promoting transparent recruitment, consistent preparation, and safe transition to practice. Strengthening these regulatory and organizational processes may improve workforce stability, patient care, and retention of internationally educated nurses.

## Introduction

Internationally educated nurses (IENs) are an important part of the U.S. healthcare workforce, especially in settings affected by nursing shortages. As the population ages and more patients need long-term care for chronic illnesses, many health systems rely on nurses educated outside the United States (Kokorelias et al., 2025; Ung et al., 2024). Many factors, including immigration pathways, credentialing and licensure requirements, and recruitment practices, shape migration (Brush & Sochalski, 2007; Mpando et al., 2025). For many nurses, migration creates opportunities for career growth and financial stability. At the same time, it may bring challenges related to cultural adjustment, workplace integration, role expectations, and unfair recruitment or employment practices (Covell et al., 2017; Pressley et al., 2022; Shaffer et al., 2020).

The pathway into U.S. practice can be difficult to navigate. IENs often move through several steps, typically beginning with recruitment and followed by visa and immigration processes, credentialing and licensure, and employer-based onboarding (Pittman et al., 2010; Shaffer et al, 2020; Walton-Roberts, 2023). *The WHO* [World Health Organization] *Global Code of Practice on the International Recruitment of Health Personnel* calls for fair, transparent, and mutually beneficial recruitment (WHO, 2010). In practice, however, the level of preparation and support for IENs is inconsistent throughout the migration process (Buchan et al., 2022, p. 13; Walton-Roberts, 2023).

Most of the literature on IENs has centered on individual and work-related experiences, such as acculturation, barriers to communication, discrimination, professional identity, and adjustment to new employment settings (Ghazal et al., 2020; Kokorelias et al., 2025; Pressley et al., 2022). These studies are valuable but generally focus on what happens to nurses after problems occur. Fewer efforts have been made to understand how systems of recruitment, preparation, certification, and onboarding coalesce along the journey to practice.

More recently, the focus has shifted from viewing adaptation as primarily the individual nurse’s responsibility to examining how leadership and organizational strategies can better support IENs. Mentorship, culturally responsive onboarding, and greater organizational accountability have been identified as promising approaches to facilitating successful transitions (Rivera et al., 2026). Other studies have suggested that fair recruitment practices, realistic workplace expectations, and coordinated transition support can improve IEN integration, job satisfaction, and retention (Kokorelias et al., 2025; Ung et al., 2024). However, these issues are often examined separately. Less is known about how recruitment, preparation, credentialing and licensure, and onboarding work together across the full pathway to practice in the United States.

To add to the complexity of incorporating IENs into the U.S. healthcare system, there are state-level differences, inequalities in healthcare structures and recruitment agencies, and significant restrictions on immigration and licensure. Newcomers may face considerable challenges in establishing themselves and gaining permanent status over time. Challenges involve visa processing, diverse onboarding procedures, and insufficient transitional support (Pressley et al., 2022).

Although these challenges are increasingly recognized, limited empirical work has examined how structural determinants operate collectively across the IEN pathway to practice in the United States. Such an understanding is critical to the development of robust policies, strategic plans, and workforce strategies to incorporate and stabilize the nursing profession amid a large shortfall. In the present study, we conceptualized IEN workforce integration as a structural process rather than an event. We created and distributed a survey that aimed to collect information on system-level actions, focusing on recruitment methods; pre-arrival preparation and support; administrative and regulatory support upon arrival; and onboarding and early workplace integration. Rather than focusing primarily on individual adaptation, this study centers on institutional and system-level structures that influence entry into practice and early workforce integration into the U.S. healthcare workforce.

## Methods

### Study design and sample

We used a cross-sectional survey design to examine the demographic and professional characteristics of IENs and the structural factors affecting their integration into the U.S. healthcare system. A convenience sampling approach was used to recruit participants through professional nursing networks and national and regional nursing associations with strong engagement among IENs. Eligibility criteria included being an IEN who had undergone, or was in the process of undergoing, transition to practice in the United States. No specific time restriction was imposed regarding when participants underwent transition to practice, allowing inclusion of nurses with varied transition experiences across career stages.

### Survey creation and data collection

Data were collected through a structured, self-administered, 68-item electronic survey. The survey was developed by members of the Philippine Nurses Association of America (PNAA; a U.S.-based nursing association) Cy Pres Task Force to capture key structural dimensions operationalized as system-level processes and conditions that influence professional entry (rather than as individual attributes of the IEN transition process), identified in the literature using categorical, ordinal, and optional open-ended response formats. Information was collected on demographic attributes (age, gender, and country of education); professional history (years of experience and area of practice); and structural components of the transition process. These components included recruitment, hiring process, predeparture orientation, credentialing and licensure, arrival support, onboarding, and initial work experiences. Participants were also provided with optional open-ended survey items to describe perceived barriers and facilitators related to transition to practice, including workplace culture adaptation, mentorship, and professional development opportunities.

To support face and content validity, survey items were reviewed by nurse leaders, IENs, and members of the PNAA Cy Pres Task Force with expertise in IEN recruitment, transition, and workforce integration before distribution.

Surveys were distributed via email on February 1, 2025, to approximately 300 IENs. No follow-up emails were sent. The electronic survey platform was configured to permit only one response per email invitation. The survey closed on April 1, 2025.

### Data analysis

Descriptive statistics were used to analyze participants’ demographic information and variables related to recruitment, preparation, arrival support, and initial workforce integration. Frequencies and percentages were computed for categorical and ordinal survey items. Because the purpose of this study was descriptive, analyses were conducted for the overall sample; subgroup comparisons between participants who had completed transition to practice and those still in the process of transition were not conducted.

### Ethical considerations

The Ethics Committee of the PNAA reviewed the study protocol. The committee determined that institutional review board approval was not required because the project used anonymous, de-identified survey data and did not collect personally identifiable information. Participation was voluntary, and electronic informed consent was obtained before participants began the survey. All responses were stored in a secure, password-protected database.

## Results

### Participant characteristics

Surveys were sent electronically to approximately 300 IENs. A total of 232 IENs completed the survey for a response rate of approximately 77%. Most participants were female (84.9%), Asian (96.6%), and held a bachelor of science in nursing degree (90.1%). Participants represented a wide age distribution, with most aged 41–55 years (44.4%) or older than 55 years (39.2%). Prior nursing experience before entering the U.S. workforce varied: 82 participants (35.3%) reported 1–3 years of clinical practice, while others reported limited prior experience or more than 5 years. Geographic location within the United States was not collected.

### Structural pathways to practice

**Fig. 1.**
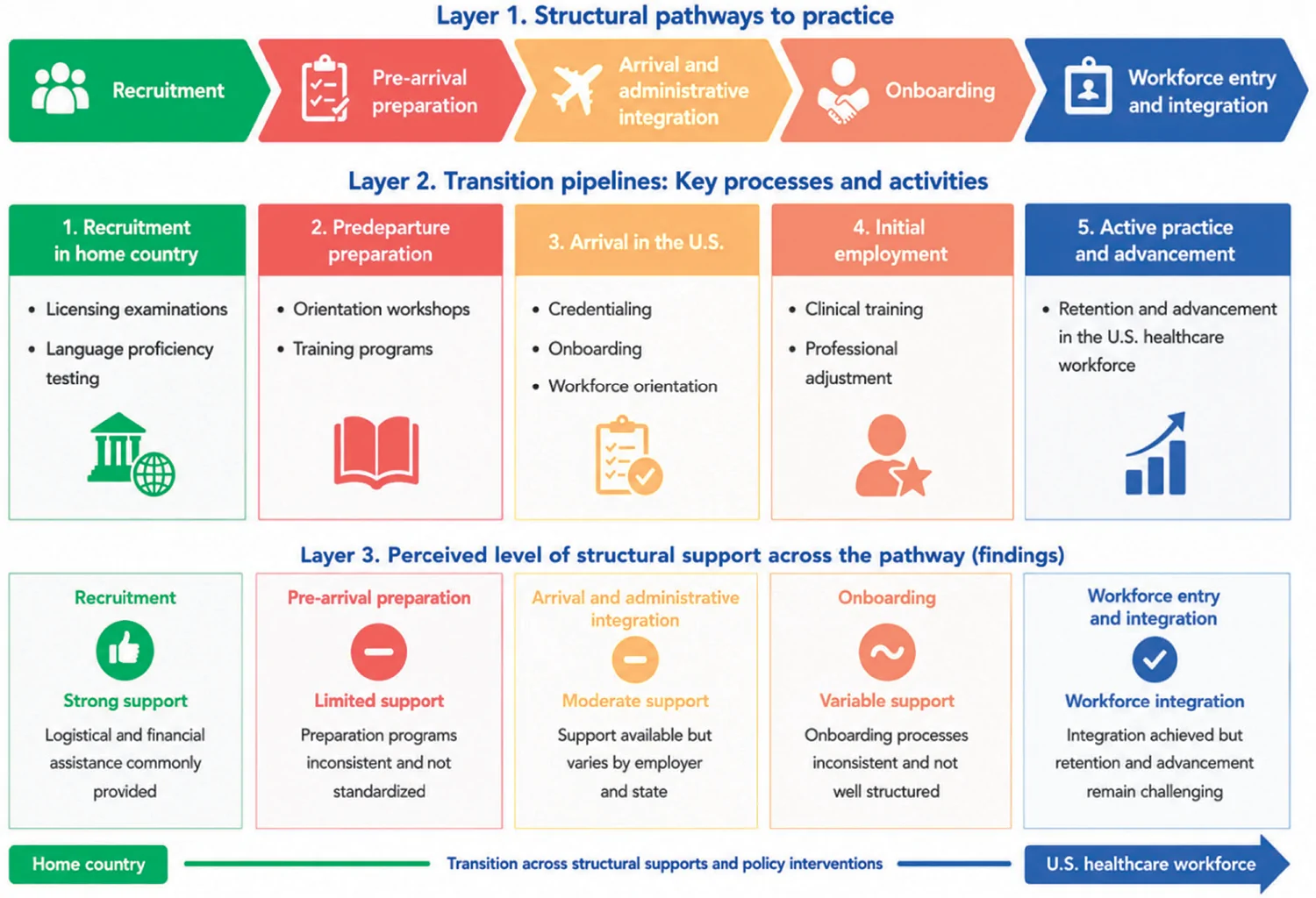
Structural pathways and transition pipelines for IENs transitioning from recruitment in their home country to active practice in the U.S. healthcare workforce. Layer 1 presents the 5 major phases of the pathway. Layer 2 outlines key processes and activities associated with each phase. Layer 3 summarizes the perceived level of structural support across the pathway based on survey findings. IEN, internationally educated nurse. This figure was developed with the assistance of ChatGPT (OpenAI) to support visualization of study findings. All content and interpretation were generated and verified by the authors.

Table 1 summarizes reported structural supports across the transition pathway organized into four phases: recruitment, pre-arrival preparation, arrival and administrative support, and onboarding. Fig. 1 presents the pathway from recruitment through workforce entry and displays the relative level of support reported across each phase.

### Recruitment pathways

Most participants (66.4%) entered the U.S. healthcare workforce through agency-assisted pathways. Other participants reported family-sponsored or independent recruitment pathways (33.6%). Reported recruitment methods included direct hire (41.7%), placement agencies (37.8%), and staffing agencies (21.9%). Fixed-term contracts of 2–3 years (66%) were the most commonly reported contractual arrangements (Table 1).

### Pre-arrival preparation

The extent of pre-arrival preparation varied. Fewer than half of participants reported receiving pre-arrival onboarding (37.7%). Logistical support was more commonly reported, including flight booking support (84.0%), airport meet-and-greet services (75.9%), travel assistance such as airline tickets, coordination of travel arrangements or relocation-related support (69.6%), arrival concierge support (58.5%), and basic welcome support (44.8%) (Table 1).

### Arrival and administrative support

Participants reported varying levels of arrival and administrative support. The most commonly reported supports were assistance with Social Security processes (69.1%), credentialing and licensure (53.6%), and banking (50.5%). Less commonly reported supports included housing assistance (38.1%), health insurance support (24.7%), and driver’s license support (16.5%) (Table 1).

### Onboarding and early workforce integration

Onboarding support also varied. Most participants reported having an assigned mentor or preceptor (72.6%) and receiving unit-specific training (71.9%). Fewer participants reported receiving clinical skills assessment (40.5%), technology training (37.3%), a formal IEN orientation program (35.3%), cultural orientation (30.1%), or ongoing follow-up support (26.7%) (Table 1).

### Workforce entry

Most participants entered the U.S. healthcare workforce as staff nurses (*n* = 208, 89.7%), and 133 participants (57.5%) reported an initial hospital-based employment setting. At the time of the survey, 146 participants (62.8%) reported current hospital employment. Most participants had been in the U.S. workforce for more than 5 years (*n* = 203, 87.7%), and 165 participants (71.3%) reported completing their initial contract.

### Perceived barriers and supports

**Fig. 2.**
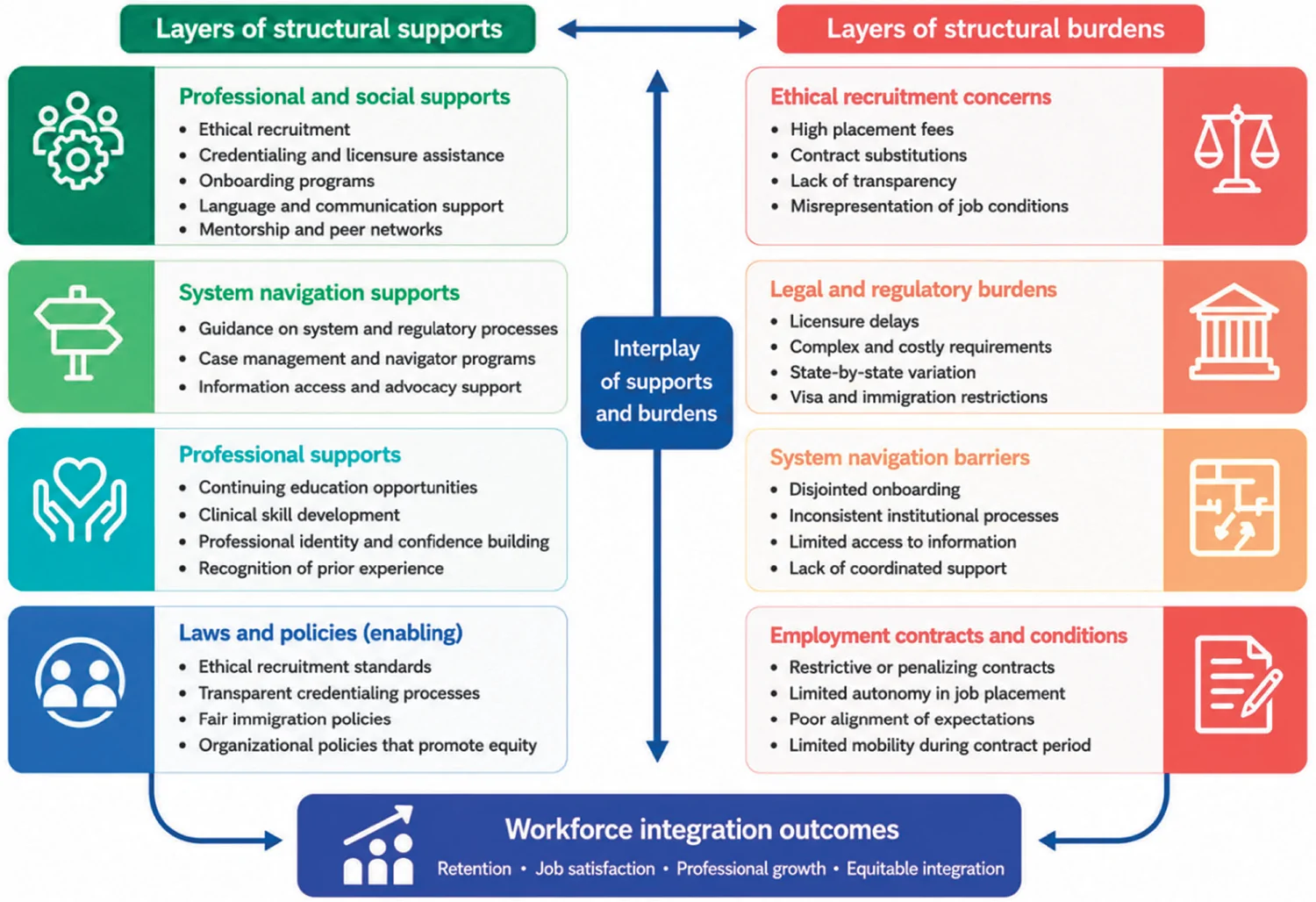
Layers of structural supports and burdens influencing the integration of IENs into the U.S. healthcare workforce. The interplay between professional, organizational, and policy-level supports and corresponding structural barriers, including regulatory, administrative, and contractual challenges that shape workforce integration, are illustrated. This figure was developed with the assistance of ChatGPT (OpenAI) to support visualization of study findings. All content and interpretation were generated and verified by the authors.

Participants also described perceived barriers and supports through optional open-ended survey responses. Common barriers included inconsistent pre-arrival preparation, limited formal IEN orientation, difficulty navigating credentialing and licensure processes, and differences in workplace culture and role expectations. Common supports included mentorship, supportive coworkers, structured onboarding, and assistance with relocation and administrative processes. Fig. 2 summarizes these perceived supports and challenges across professional, organizational, and policy levels.

## Discussion

This study examined the steps IENs move through as they enter the U.S. healthcare workforce. The findings show that support is not consistent across these steps. Logistical support, such as travel assistance and airport reception, was reported more often than formal pre-arrival preparation, cultural orientation, or ongoing follow-up. This pattern suggests that many systems are set up to help nurses arrive and begin work, but fewer systems are in place to support their full transition into practice.

### Alignment with existing literature on IEN integration

The diversity of recruitment pathways and preparation before arrival aligns with previous research indicating that IEN migration involves multiple routes, including agency-based and direct-hire channels (Brush, 2010; Mpando et al., 2025; Organisation for Economic Co-operation and Development, 2016). These approaches may differ in the amount of information, preparation, and support that nurses receive. Logistical support (i.e., trip coordination) was often reported in this study, but more formal training for U.S. clinical practice and workplace requirements was less consistent. This study supports prior concerns regarding ethical recruitment, preparedness for practice, and the long-term sustainability of the nursing workforce (Buchan et al., 2022, p. 13; Pittman et al., 2010).

Onboarding was similar. Mentorship and unit-level training were reported more often than formal IEN orientation, cultural orientation, or ongoing follow-up. This finding aligns with prior research indicating that fragmented onboarding can result in role strain, longer adjustment periods, and difficulties with professional integration (Cubelo et al., 2024; Ghazal et al., 2020). It also supports previous work suggesting that IEN integration is enhanced when orientation, mentorship, and organizational support are planned and sustained over time (Covell et al., 2017; Kokorelias et al., 2025; Pressley et al., 2022).

### Enhancing evidence: A structural pathways approach

Much of the existing literature on IENs has focused on individual experiences, such as acculturation, discrimination, and workplace adaptation (Alibudbud, 2026; Turnbull et al., 2024). These experiences are important, but they do not fully explain why some nurses receive stronger support than others. The present study adds to the literature by focusing on the systems around the IEN, including recruitment, preparation, credentialing and licensure, arrival support, and onboarding.

Viewing integration as a pathway helps show how gaps in one stage may affect later stages. For example, limited pre-arrival preparation may make onboarding more difficult, especially when cultural orientation and ongoing follow-up are also limited. This understanding is consistent with systems-level research showing that workforce integration is shaped not only by the nurse’s ability to adapt but also by policies, organizational practices, and regulatory requirements (Kokorelias et al., 2025; Ung et al., 2024).

### Structural fragmentation and regulatory implications

A key finding is that support was uneven across the pathway to practice. Nurses were more likely to receive help with travel and arrival than with pre-arrival onboarding, formal IEN orientation, cultural orientation, or follow-up after starting work. Fig. 2 shows how support and challenges can occur at different levels, including in the workplace, by the employer, and in the broader policy environment.

Our findings suggest a need for more collaboration between recruitment agencies, employers, and regulatory bodies. Although The WHO Global Code of Practice promotes ethical recruitment and mutual benefit, these ideals can be difficult to implement (Buchan et al., 2022, p. 13; WHO, 2010). In the United States, credentialing and licensure requirements, visa procedures, and employer-based onboarding can also affect the smooth transition of IENs into practice. Nursing regulatory bodies play an important role in establishing standards for licensure, public protection, and entry into practice. Working with employers and recruitment agencies can help promote more consistent preparation, more ethical recruitment, and a safer transition to practice for internationally educated nurses (Pittman et al., 2010; Xu & Kwak, 2007).

At present, much of the support IENs receive appears to depend on the employer or agency involved. This means that IENs may have very different experiences depending on who recruited them, where they are placed, and what support is available. These differences raise concerns about fairness, readiness for practice, and retention.

### Resilience in the context of structural variability

Even with uneven support, many participants stayed in the U.S. workforce and worked beyond their initial contracts. This process is consistent with prior research showing that IENs, including Filipino nurses, have made long-standing contributions to the global nursing workforce (Brush, 2010; Montayre et al., 2018).

However, retention does not mean that current support systems are enough. Some nurses may succeed despite limited support. When preparation and onboarding are inconsistent, nurses may have to carry more of the burden themselves. Over time, this may affect job satisfaction, professional growth, and nurses’ decisions to stay with an employer or organization (Covell et al., 2017; Pressley et al., 2022).

### Implications for regulatory frameworks and workforce strategy

The findings of the present study point to a few practical steps. First, recruitment and workforce integration need to be better connected. Preparing IENs for practice should start before arrival and continue during onboarding and early employment.

Second, employers should consider onboarding programs designed specifically for IENs. These programs should include clinical orientation, cultural orientation, mentorship, and follow-up after placement. Leadership support and organizational accountability are also important in helping IENs transition successfully (Rivera et al., 2026).

Third, recruitment agencies, employers, and regulatory bodies need to work more closely together. Clear expectations for recruitment, preparation, credentialing and licensure, and onboarding may help reduce differences in support across settings. Better coordination may also support ethical recruitment, smoother transition to practice, job satisfaction, and retention.

### Strengths and limitations

The study has several strengths. It draws on a national sample of IENs and examines several stages of the transition process within a single framework. By focusing on structural supports rather than on individual adaptation alone, this study offers a systems-level view of how IENs enter the U.S. workforce. The study also achieved a high survey response rate, which strengthens confidence in the findings.

Several limitations should also be noted. This study used convenience sampling, so the findings may not represent all IENs in the United States. Also, the sample was predominantly Asian and included many Filipino nurses, reflecting an important group within the global nursing workforce, but potentially limiting the extent to which the findings apply to other IEN groups. Additionally, the data were self-reported, so recall bias is possible, especially given the lack of a time limit on when participants began or completed their transition to practice. U.S. geographic location was not collected, and subgroup analyses comparing nurses who had completed transition with those still undergoing transition were not performed. In addition, the survey was conducted before recent changes in U.S. immigration and visa policies. Future studies should examine how evolving immigration requirements and workforce policies may influence the recruitment, transition, and integration of internationally educated nurses. Finally, because this was a cross-sectional study, the findings describe patterns of support but cannot establish changes over time or causal relationships.

## Conclusion

The findings of this study show that IENs move through a complex pathway as they enter the U.S. healthcare workforce. While many participants reported receiving support with recruitment and relocation, assistance during pre-arrival preparation, onboarding, and early workforce integration was less consistent. These differences suggest opportunities to strengthen coordination across recruitment agencies, employers, and regulatory bodies. More structured preparation and onboarding may help support smoother transitions, improve job satisfaction, and strengthen retention. Future research should continue to examine how systems and policies can better support IENs throughout the transition-to-practice process.

## Figures and Tables

**Table 1:** Structural pathways and support for IENs by transition phase (*N* = 232).

Phase and domain	*n*/*N* (%)
Recruitment	
Agency-assisted pathway	142/214 (66.4%)
Family/independent pathway	72/214 (33.6%)
Direct hire	63/151 (41.7%)
Placement agency	57/151 (37.8%)
Staffing agency	33/151 (21.9%)
Contract length (2–3 y)	105/159 (66%)
Pre-arrival preparation	
Pre-arrival onboarding provided	61/162 (37.7%)
Travel assistance	87/125 (69.6%)
Flight booking support	89/106 (84.0%)
Airport meet and greet	88/116 (75.9%)
Arrival concierge	62/106 (58.5%)
Basic welcome support	52/116 (44.8%)
Arrival and administrative support	
Social Security assistance	67/97 (69.1%)
Licensure processing support	52/97 (53.6%)
Banking support	49/97 (50.5%)
Housing assistance	37/97 (38.1%)
Health insurance support	24/97 (24.7%)
Driver’s license support	16/97 (16.5%)
Onboarding and integration	
Assigned mentor/preceptor	111/153 (72.6%)
Unit-specific training	110/153 (71.9%)
Clinical skills assessment	62/153 (40.5%)
Technology training	57/153 (37.3%)
Cultural orientation	46/153 (30.1%)
Formal IEN orientation program	54/153 (35.3%)
Ongoing follow-up support	35/131 (26.7%)

**Note:** IEN, internationally educated nurse. Percentages may not total 100% owing to missing data and multiple response options.
